# Discovery of a Novel, Isothiazolonaphthoquinone-Based Small Molecule Activator of FOXO Nuclear-Cytoplasmic Shuttling

**DOI:** 10.1371/journal.pone.0167491

**Published:** 2016-12-09

**Authors:** Bastien Cautain, Francisco Castillo, Loana Musso, Bibiana I. Ferreira, Nuria de Pedro, Lorena Rodriguez Quesada, Susana Machado, Francisca Vicente, Sabrina Dallavalle, Wolfgang Link

**Affiliations:** 1 Fundacion MEDINA, Parque Tecnológico Ciencias de la Salud, Granada, Spain; 2 DeFENS Department of Food, Environmental and Nutritional Sciences, Università di Milano, Italy; 3 Centre for Biomedical Research (CBMR), Gambelas Campus, Faro, Portugal; 4 Regenerative Medicine Program, Department of Biomedical Sciences and Medicine, University of Algarve, Campus de Gambelas, Faro, Portugal; Hungarian Academy of Sciences, HUNGARY

## Abstract

FOXO factors are tumour suppressor proteins commonly inactivated in human tumours by posttranslational modifications. Furthermore, genetic variation within the FOXO3a gene is consistently associated with human longevity. Therefore, the pharmacological activation of FOXO proteins is considered as an attractive therapeutic approach to treat cancer and age-related diseases. In order to identify agents capable of activating FOXOs, we tested a collection of small chemical compounds using image-based high content screening technology. Here, we report the discovery of LOM612 (compound 1a), a newly synthesized isothiazolonaphthoquinone as a potent FOXO relocator. Compound 1a induces nuclear translocation of a FOXO3a reporter protein as well as endogenous FOXO3a and FOXO1 in U2OS cells in a dose-dependent manner. This activity does not affect the subcellular localization of other cellular proteins including NFkB or inhibit CRM1-mediated nuclear export. Furthermore, compound 1a shows a potent antiproliferative effect in human cancer cell lines.

## Introduction

The mammalian forkhead transcription factors of the O class (FOXOs) consists of four proteins, FoxO1, FoxO3, FoxO4 and FoxO6 [1,2]. FoxOs have a characteristic forkhead box DNA binding domain and bind as monomers to their consensus DNA binding sites [3]. FOXO proteins function as transcriptional regulators in the cell nucleus and activate the transcription of genes that are involved in numerous biologically relevant processes such as metabolism, differentiation, proliferation, longevity, and apoptosis [4]. FoxOs are key components of an evolutionary conserved pathway downstream of insulin and insulin-like growth factor receptors. Posttranslational modifications are thought to be the main mechanism to regulate their activity [5]. The Serine/Threonine kinase AKT phosphorylates FOXO proteins at three conserved consensus sites, which leads to conformational changes that facilitate CRM-1- mediated nuclear export abolishing FOXO-dependent gene transcription [6]. Under stress conditions or in the absence of growth or survival factors, when the PI3K/AKT pathway is inhibited, FOXO proteins translocate to the cell nucleus, where their transcriptional functions can be executed [5,7]. FOXO factors have been found to be inactivated in the majority of human cancers, owing to the overactivation of the phosphoinositide 3-kinase (PI3K)/AKT pathway [5,7] and have been established as *bona fide* tumor suppressors [8]. Accordingly, FOXOs are the major downstream transcriptional mediators of the PI3K/AKT pathway after PI3K inhibition [9]. Furthermore, FoxO proteins are known to determine longevity in a wide range of metazoans including humans and are indispensable for lifespan extension in response to dietary restriction regimens [10]. Recent studies have found that FOXO3A is associated with human longevity in Japanese-Americans from Hawaii, Italians, Ashkenazi Jews, Californians, New Englanders, Germans and Han Chinese [11]. The importance of FOXO proteins in tumor suppression and aging renders them compelling targets in the quest for therapeutic agents against cancer and to slow down the aging process. Several assays have been developed to monitor the subcellular localization of FOXO factors [12–14]. In order to identify chemical agents with the capacity of activating FOXO, we chose to employ a high content imaging approach to monitor the nucleocytoplasmic translocation of a GFP-FOXO3a fusion protein in U2OS cells. In this study, we identified and characterized a novel small FOXO relocator molecule.

## Materials and Methods

### Compounds

The compounds used for primary screening were obtained within the framework of COST Actions CM1106 (Chemical Approaches to Targeting Drug Resistance in Cancer Stem Cells) responsible for generating a compound collection for further biological evaluation. The COST CM1106 compound collection contains synthetic molecules and natural compounds. LY294002 and Leptomycin B were purchased from Calbiochem.

### Synthesis of compounds 1a-c

#### General information

All reagents and solvents were reagent grade or were purified by standard methods before use. Melting points were determined in open capillaries and are uncorrected. Solvents were routinely distilled prior to use; dry methylene chloride was obtained by distillation from phosphorus pentoxide. All reactions requiring anhydrous conditions were performed under a positive nitrogen flow, and all glassware were oven dried. Column chromatography was carried out on flash silica gel 60 (230–400 mesh). Analytical and preparative thin-layer chromatography (TLC) was conducted on TLC plates (silica gel 60 F254, aluminium foil) and spots were visualized by UV light and / or by means of dyeing reagents. NMR spectra were recorded at 300 MHz. Chemical shifts (δ values) and coupling constants (J values) are given in ppm and Hz, respectively. Compound *N*,*N*-dibenzyl-urea (**2a**) was prepared according to literature procedure [15]. Compound **3a** was prepared according to literature procedure [16]. Compound **3c** was prepared according to literature procedure [17].

#### 3-Dimethylamino-naphtho[2,3-d]isothiazole-4,9-dione (1a)

To a stirred solution of naphthoquinone (2.5 g, 16mmol) in xylene (20mL) compound **3a** (777mg, 5.3mmol) was added and the resulting mixture was heated to reflux for 2h. The solvent was removed under reduced pressure and the residue was purified by preparative chromatography in hexane/acetone 19:1 to afford compound **1a** (314 mg, 23%): mp: 170°C; ^1^H-NMR (300 MHz, CDCl_3_) δ: 8.31–8.11 (m, 2H), 7.89–7.77 (m, 2H), 3.12 (s, 6H). ^13^C-NMR (75 MHz, CH_3_OH-d_4_)) δ: 178.02, 177.49, 168.69, 167.42, 134.93, 134.87, 133.29, 132.58, 127.81, 126.88, 124.77, 41.90 (× 2). Anal. calcd for C_13_H_10_N_2_O_2_S: C 60.45, H 3.90, N 10.85, found: C 60.37, H 3.91, N 10.87.

#### 5-Dibenzylamino-[1,3,4]oxathiazol-2-one (3b)

To a suspension of *N*,*N*-dibenzyl-urea (1 g, 4.16mmol) in acetonitrile (12mL), chlorocarbonylsulphenyl chloride (117μL, 1.38mmol) was added and the mixture was stirred 20 h at room temperature (RT). The solvent was removed under reduced pressure and the residue was purified by preparative chromatography in hexane/ethyl acetate 9:1 to obtain compound **3b** as a pale yellow oil (254mg, 62%): ^1^H-NMR (300 MHz, acetone-*d*_6_) δ: 7.49–7.21 (m, 10H), 4.6 (s, 4H).

#### 3-(Benzyl-phenyl-amino)naphtho[2,3-d]isothiazole-4,9-dione (4)

To a stirred solution of naphthoquinone (402mg, 2.55mmol) in xylene (5mL), compound **3b** (254mg, 0.85mmol) was added and the resulting mixture was heated to reflux for 3 h. The solvent was removed under reduced pressure, and the residue was purified by preparative chromatography in toluene/ethyl ether 200:1 resulting in compound **4** (219 mg, 63%): mp: 93°C; ^1^H-NMR (300 MHz, CDCl_3_) δ: 8.32–8.19 (m, 2H), 7.88–7.68 (m, 2H), 7.40–7.15 (m, 10H), 4.71 (s, 4H). ^13^C-NMR (75 MHz, CH_3_OH-d_4_) δ: 177.8, 177.7, 167.8, 167.6, 137.70 (× 2), 135.0, 134.85, 133.4, 132.5, 128.4 (× 4), 128.3 (× 4), 127.9, 127.2 (× 2), 126.9, 125.27, 54.52 (× 2). Anal. calcd for C_25_H_18_N_2_O_2_S: C 73.15, H 4.42, N 6.82, found: C 73.34, H 4.43, N 6.80.

#### 3-Amino-naphtho[2,3-d]isothiazole-4,9-dione (1b)

To a stirred solution of compound **4** (20mg, 0.05mmol) in acetonitrile/H_2_O 9:1 (2mL), cerium ammonium nitrate (133mg, 0.25mmol) was added and the resulting mixture was stirred at room temperature for 1 h. The reaction mixture was concentrated, diluted with cold water and extracted three times with ethyl acetate. The combined extract was washed with brine and dried on Na_2_SO_4_. The crude was purified by preparative chromatography in hexane/ethyl acetate 4:1 to generate compound **1b** (62mg, 89%): mp: 238°C; ^1^H-NMR (300 MHz, DMSO-*d*_6_) δ: 8.25–8.09 (m, 2H), exchangeable with D2O). 13C-NMR (75 MHz, DMSO-d6) δ: 179.5, 177.7, 165.5, 164.7, 135.7, 134.7, 134.0, 133.9, 127.6, 127.3, 120.9. Anal. calcd for C_11_H_6_N_2_O_2_S: C 57.38 H 2.63, N 12.17, found: C 57.42, H 2.62, N 12.19.

#### 3-Phenyl-naphtho[2,3-d]isothiazole-4,9-dione (1c)

To a stirred solution of naphthoquinone (280mg, 1.77mmol) in xylene (2.5mL), compound **3c** (105mg, 0.59mmol) was added and the resulting mixture was heated to reflux for 4 h. The solvent was removed under reduced pressure, and the residue was purified by preparative chromatography in toluene/ethyl ether 200:1 to generate compound **4** (65mg, 38%): mp: 218°C; ^1^H-NMR (300 MHz, DMSO-*d*_6_) δ: 8.24–8.12 (m, 2H), 8.04–7.88 (m, 2H), 7.80–7.70 (m, 2H), 7.58–7.44 (m, 3H). 13C-NMR (75 MHz, DMSO-d6) δ: 178.6, 178.2, 169.6, 167.5, 136.0, 134.9, 134.8 (× 2), 134.0, 133.0, 130.5, 130.1 (× 2), 128.6 (× 2), 128.1, 127.3. Anal. calcd for C_17_H_9_NO_2_S: C 70.09, H 3.11, N 4.81, found: C 70.21, H 3.11, N 4.80.

### Cell culture

The human osteosarcoma cell line U2OS, the breast cancer cell line MCF7, the melanoma cell line A2058, the neuroblastoma cell line SH-SY5, the human liver cancer cell line HepG2 and the human liver epithelial cell line THLE2 were obtained from the American Type Culture Collection (Manassas, VA) and cultured in Dulbecco’s modified Eagle’s medium, supplemented with 10% fetal bovine serum (Sigma) and penicillin-streptomycin. Cell cultures were maintained in a humidified incubator at 37°C with 5% CO2 and passaged when confluent using trypsin/EDTA. Stable cell lines U2nesRELOC and U2foxRELOC cells have been generated as described previously [13,14,18,19]. GFP-NFAT was kindly provided by L. Gerace.

### Cytotoxic activity of the compounds

The cytotoxic activity of the different compounds was tested against four tumor cell lines in the MTT colorimetric assay. Cells were seeded at a concentration of 1× 10^4^ cells/well in 200μl culture medium and incubated at 37°C in 5% CO2. After 24 hours, when the monolayer formed, the medium was replaced with a final volume of 200μl of new medium with tested compounds or controls were added to the plates. Cells were treated with eight 2-fold serial dilutions of each compound spanning concentrations from 50μM to 0.39μM in 1% DMSO final. Controls are on the first and the last columns of the plates. On the first column, methyl methanesulfonate (MMS) acts as a positive control and DMSO as a negative control. On the last column there are four points of rotenone and doxorubicin with an initial concentration of 10mM and dilution ½. When compounds and controls were added, plates were incubated at 37°C in 5% CO2 incubator for 72 hours. After this time, MTT solution was prepared at 5 mg/ml in PBS 1X and then diluted at 0,5mg/ml in MEM without phenol red. The sample solution in wells was flicked off and 100μl of MTT dye was added to each well. The plates were gently shaken and incubated for 3 hours at 37°C in 5% CO2 incubator. The supernatant was removed and 100μl of DMSO 100% was added. The plates were gently shaken to solubilize the formed formazan. The absorbance was measured using a multireader Victor^™^ at a wavelength of 570nm.

### FOXO translocation assay

The U2foxRELOC system is a FOXO translocation assay that has been previously established [13]. Briefly, cells were seeded at a density of 20,000 cells per well into black wall clear bottom 96-well microplates (greiner bio-one; Frickenhausen, Germany). After 12 h of incubation at 37°C with 5% CO2, 2μl of each test compound (1mM stock) was transferred to the assay plates. Cells were incubated with the different compounds for 30 minutes. Cells were fixed with 100% methanol, and the nucleus stained with DAPI (Invitrogen). Cells were washed twice with 1x phosphate-buffered saline (PBS) and stored at 4°C before analysis. All liquid handling for compound treatment, washing, fixing, and staining steps were performed by a robotic workstation (cell culturing robot, Select T, TAP Biosystems). All experiments were performed in triplicate. For dose response experiments cells were cultured as described above and exposed for one hour to equal volumes of test compounds (2μl). IC50 values were calculated as being the inhibitor concentration that increases nuclear accumulation of the reporter protein by 50% using Genedata Screener software (Genedata AG, Switzerland). Cells were treated with eight 2-fold serial dilutions of each compound spanning concentrations from 50μM to 0.39μM in 1% DMSO final.

### Nuclear export assay

The U2nesRELOC assay, a previously established nuclear export assay[14] is based on the reporter construct pRevMAPKKnesGFP. pRevMAPKKnesGFP carries the NES from MAPK kinase (MAPKK) (or MEK) cloned between the BamHI and AgeI sites of pRev(1.4)-GFP, sandwiched between the Rev and the green fluorescent protein (GFP) coding sequences.

### Image acquisition and processing

The BD Pathway 855 High Content Bioimager (BD Biosciences; San Jose, CA) was used for automated image acquisition. Acquired images were processed using AttoVision software (BD Biosciences; San Jose, CA). The Bioimager was equipped with a 488/10 nm enhanced GFP (EGFP) excitation filter, a 380/10 nm DAPI excitation filter, a 515LP nm EGFP emission filter, and a 435LP nm DAPI emission filter. Images were acquired in the DAPI and GFP channels of each well using a 10 X dry objective. The plates were exposed 0.066 ms (Gain 0) to acquire DAPI images and 0.85 ms (Gain 30) for GFP images. Cells were stained with DAPI to facilitate microscope autofocus and to aid in the image segmentation. An image algorithm based on a local threshold was applied to allow for the cell nucleus segmentation. Our segmentation strategy assumes that the cell’s cytoplasm surrounds the nucleus. Consequently, cytoplasmic fluorescence intensity is calculated from all the pixels within a circumferential ring surrounding the nuclear ring mask. The width of the ring was defined to be small enough to avoid ambiguities due to irregular cell shape. Based on the definition of cell compartments, the nuclear and cytoplasmic levels of GFP fluorescence were quantified.

### Data analysis

The BD Pathway Bioimager generates standard text files that were imported into the BD Image Data Explorer data analysis software. The nuclear/cytoplasmic fluorescence intensity ratios were determined by dividing the fluorescence intensity of the nucleus by the cytoplasmic fluorescence intensity. A threshold ratio greater than 1.8 was employed to define nuclear accumulation of fluorescent signal for each cell. Based on this procedure, we calculated the percentage of cells per well displaying nuclear translocation or inhibition of nuclear export. Compounds that induced a nuclear accumulation of the fluorescent signal greater than 60% of that obtained from wells treated with 4 nM LMB (LC Laboratories, Woburn, MA, USA) were considered to be hits. We also monitored nuclear shrinkage known to be associated with apoptosis by analyzing the number of “nuclear vertices”. The term “nuclear vertices” refers to the number of pixels that lie on the nuclear boundary. In order to estimate the quality of the HCS assay, the Z' factor was calculated by the equation: Z`= 1 –[(3 × std. dev. of positive controls) + (3 × std. dev. of negative controls) / (mean of positive controls)—(mean of negative controls)] as previously described by Zhang and colleagues[20].

### Immunofluorescence analysis by confocal microscopy

U2OS cells were cultured as described above and exposed for 30 min at 1.5μM LOM612 (compound 1a). The cells were fixed with 100% methanol (5 min) and then permeablilized and blocked for 1hour with 1%BSA / 10% normal goat serum / 0.3M glycine in 0.1% PBSTween. The cells were then stained with FOXO3a antibody (Cell Signaling Technology^®^ #2497S), FOXO1 antibody (Cell Signaling Technology^®^ #2880) or NFKB2 p100/p52 Antibody (Cell Signaling Technology^®^ #4882) overnight at 4°C followed by DyLight^®^594 donkey anti-rabbit (Abcam ab96921) secondary antibody for 1h. DAPI was used to counterstain the cell nuclei at a concentration of 1.43 μM. Samples were imaged at 22°C using 63× water immersion lenses on a Leica SPE confocal imaging system. Images were obtained using Leica software.

### qPCR analysis

mRNA from U2OS cell line was isolated using RNAeasy (QIAGEN) kit and reverse transcribed using NZY First-strand cDNA Synthesis kit (NZYtech, PT). qPCR was done in technical triplicate for each sample. qPCR reaction was carried out using Lumino Ct SYBR Green qPCR ReadyMix (Sigma). All qPCR results are representative of two separate experiments. Data analysis was carried out using the ΔCt method. Comparisons were made using the unpaired Student’s t test. Values represent the mean ± standard error of the mean (SEM) and are represented as error bars. Statistical significance as indicated.

## Results

### Identification of specific FOXO relocator compounds

**Fig 1:**
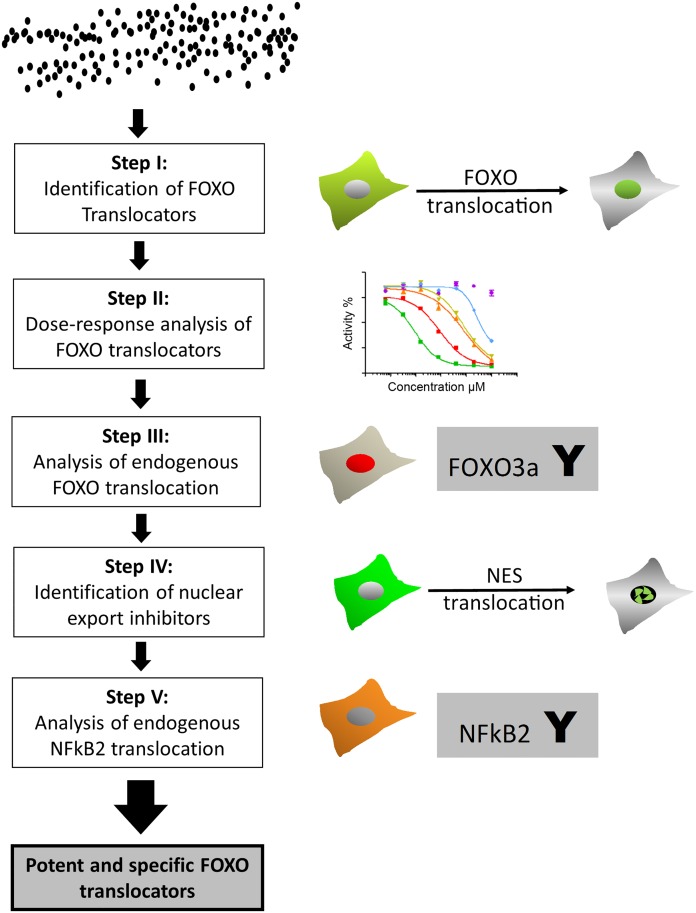
Screening strategy to identify specific and potent FOXO translocator compounds. As a primary filter a FOXO translocation assay has been used, followed by dose response analysis of the confirmed hit compounds. The effect of the hit compounds on endogenous FOXO proteins has been assessed by immunohistochemistry. Then inhibitors of the general nuclear export machinery were excluded using a nuclear export assay. Finally, the effect on other transcription factors has been determined by immunohistochemistry using a NFKB2 specific antibody.

In order to identify specific FOXO relocator compounds we designed a sophisticated screening strategy (Fig 1). As the primary filter, we used a cellular imaging assay that follows the intracellular location of FOXO proteins which we have developed previously [13] and used with success to discover anti-cancer drug candidates, including PI3K inhibitors [21–25]. The rapid kinetics of the assay allowed us to reduce the incubation time and minimize possible toxic effects that might interfere with the analysis. Furthermore, this image-based high-throughput strategy provides a filter for adequate solubility, permeability, and stability in a cellular context and enables compounds that produce artifacts or cytotoxicity to be identified on a single cell basis [19,26]. The U2OS cell line that stably express a green fluorescent protein (GFP)-tagged FOXO3a were seeded in 96-well assay plates and treated with different compounds at a final concentration of 10μM for 30 min. A total of 544 compounds were screened and their ability to induce GFP-FOXO3a reporter protein nuclear translocation was assessed. The nuclear accumulation of GFP induced by Leptomycin B (LMB), an inhibitor of the CMR-1-dependent nuclear export that covalently binds to a single cysteine residue of the CRM1 protein, was used as a reference and defined as 100% activity. 0.5% DMSO was used as a vehicle control. Primary hits were defined as those compounds that have an activity greater than 60%. Several compounds matched the criteria and were cherry-picked to confirm the activity and reproducibility. Among the confirmed hit compounds we identified compound **1a** (LOM612), a newly synthesized isothiazolonaphthoquinone. The isothiazolequinones are an extremely rare class of compounds. To the best of our knowledge, only two natural products, pronqodine A [27] and aulosirazole [28] and a few synthetic compounds [29,30] have been reported in the literature so far. Therefore, this skeleton can be considered as a very attractive target for biological evaluation, offering the possibility of preparing products with several points of diversity for decoration.

**Fig 2:**
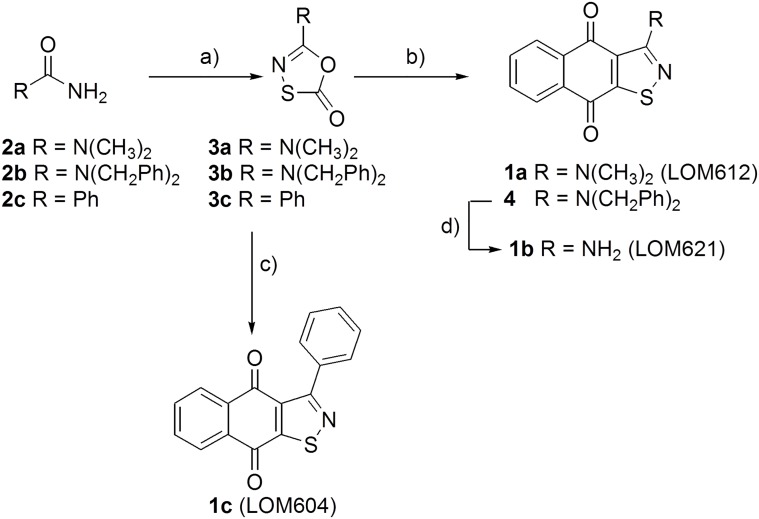
Synthesis of compounds 1a-c (LOM612/621/604). (a) chlorocarbonylsulphenyl chloride, *N*, *N*-dimethylurea or *N*,*N*-dibenzylurea, acetonitrile, 2h RT for **3a**: 84%, **3b**: 62%; chlorocarbonylsulphenyl chloride, benzamide, toluene, 3h, reflux **3c**: 70%; (b) 1,4-naftochinone, **3a**, xilene, 80°C, 3h, 23%; 1,4-naftochinone, **3b**, xylene, 80°C, 3h, 63%; (C) 1,4-naftochinone, **3c**, xilene, 8h, reflux, 3h, 38%; d) CAN, CH_3_CN: H_2_O 9:1, 1h, RT, 89%.

Compound **1a**, together with its analogues **1b-c,** were prepared according to the two-step procedure reported in Fig 2. The 1,3,4-oxathiazol-2-ones **3a** and **3b** [31] were synthesized in good yield by reaction of *N*,*N*-dimethylurea or *N*,*N*-dibenzylurea urea and chlorocarbonylsulphenyl chloride respectively. Then, the 1,3-dipolar cycloaddition between the nitrile sulfide generated *in situ* by termal decarboxylation of **3a** and **3b** [32] and the naphthoquinone gave isothiazolonaphthoquinones **1a** and **4**. The intermediate **4** was then treated with cerium ammonium nitrate (CAN) in acetonitrile–water [33] at room temperature and compound **1b** was obtained in high yield. Following the same synthetic strategy isothiazolonaphthoquinones **1c** was obtained starting from 1,3,4-oxathiazol-2-ones **3c.**

**Fig 3:**
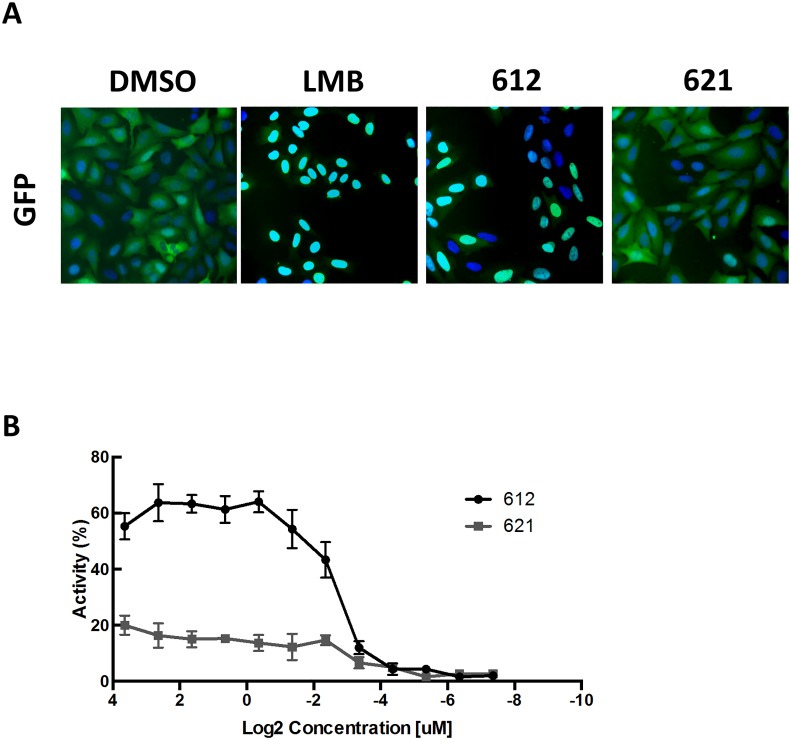
Primary screening identifies compounds capable of inducing FOXO translocation. (A) U2fox RELOC cells were treated either with DMSO, 4nM LMB, 10μM of compound LOM 612 or compound LOM 621 for 30 min. representative images are shown. (B) Dose-response relationship of the nuclear–cytoplasmic shuttling of FOXO following LOM 612 treatment. LOM612 induces nuclear translocation in a dose dependent manner. Represented is the percentage of cells with more GFP fluorescence accumulation in nucleus than in cytoplasm. Results represent the mean of three independent experiments.

Interestingly, in our experiments, compound **1b** (LOM621) which differs from compound **1a** LOM612 in the amino group by the replacement of two methyl groups by two hydrogens (Fig 2) did not induce nuclear accumulation of fluorescent FOXO reporter protein at a 10μM concentration (Fig 3A). Similarly, compound **1c**, carrying a phenyl ring in place of the amino group linked to the isothiazole scaffold also failed to produce nuclear translocation of the reporter protein (data not shown) and was not further analyzed. These results suggest that the mode of action of LOM612 depends on stringent structural requirements.

### Dose–response analysis of FOXO translocation

We next sought to determine the half maximal effective concentration (EC_50_) value of LOM612 compound. The FOXO reporter cells were cultured as described previously and treated with twelve different concentrations of LOM612, LOM621 and LMB for 30 min maintaining the transferred volume constant. Fig 3B shows that LOM612 potently activates nuclear translocation of FOXO with an EC_50_ value of 1.5μM. The EC_50_ value of the reference compound LMB was 2.3nM (data not shown) in these experiments. In contrast, LOM621 failed to induce the accumulation of GFP-FOXO in the nucleus (Fig 3A and 3B). None of the tested concentrations of LOM621 affected the nuclear localization of FOXO indicating that a small difference in the chemical structure is able to abolish the effect of these quinone derivatives on FOXO transcription factors. Taken together, these results show that LOM612 potently induces FOXO translocation in a dose-dependent manner.

### Analysis of the translocation of endogenous FOXO upon LOM612 exposure

**Fig 4:**
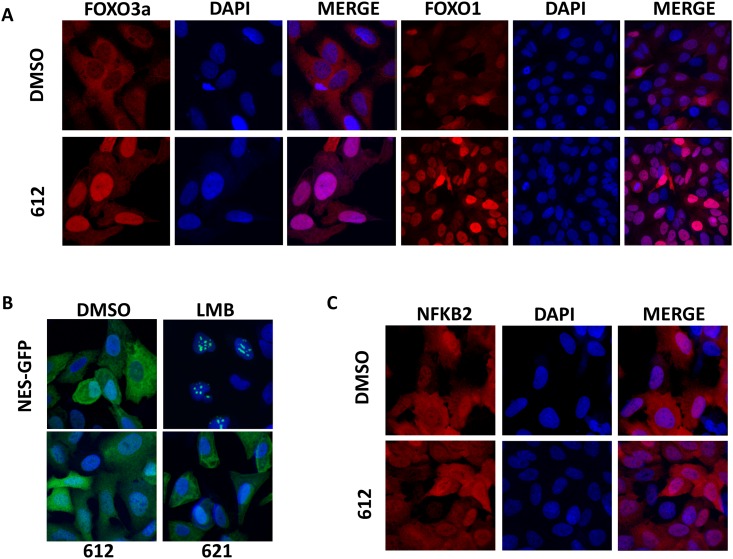
LOM612 specifically induces the nuclear translocation of endogenous FOXO proteins. (A) Compound LOM612 induces the nuclear translocation of endogenous FOXO3a and FOXO1 protein detected by using a specific antibodies after 30 min of drug exposure. (B) LOM612 does not inhibit the nuclear export. U2OS cells stably expressing nuclear export signal (NES) (Rev-NES-EGFP) reporter were treated with DMSO, LMB, LOM612 and LOM621 for 30 min. (C) LOM612 does not induce nuclear translocation of endogenous nuclear factor (NF)–κB2 protein. Representative images of the compound-treated cells using a Leica SPE confocal imaging system. Cells were seeded automatically at appropriate density in 96-well black-wall clear-bottom tissue culture plates and allowed to attach overnight. Cells were then treated with compounds for 30 min before paraformaldehyde (Rev-NES-EGFP) or methanol (FOXO3a, NFKB2) fixation and DAPI staining.

As the subcellular localization of a GFP tagged FOXO reporter protein might not reflect the nucleocytoplasmic trafficking of the endogenous protein, we performed immunohistochemical detection of FOXO3a protein in U2OS osteosarcoma cells using a specific antibody. We analyzed if exposure to LOM612 affects also the subcellular localization of endogenous FOXO proteins. As depicted in Fig 4A, LOM612 very efficiently induced translocation of endogenous FOXO3a and FOXO1. As expected, exposure of U2OS cells to LOM621 didn’t show nuclear accumulation of FOXO3a and FOXO1 proteins (data not shown). These data suggests that LOM612 can induce the nuclear translocation of FOXO factors in their physiological context. In addition, we found that the expression of the FOXO target genes p27 and FasL were increased upon exposure to LOM612 (S1 Fig).

### Analysis of the nuclear export upon LOM612 exposure

The intracellular localization of FOXO factors is regulated by posttranslational modifications, which can lead to their nuclear export via the nuclear export receptor CRM-1. CRM-1 is the best studied nuclear export receptor responsible for the nuclear export of proteins that contain a nuclear export signal (NES). Accordingly, compounds which interfere with CRM-1-mediated nuclear export induce the accumulation of NES-bearing proteins including FOXO factors. In order to determine if LOM612 triggers nuclear localization of FOXO by inhibiting CRM1, we used a high content screening approach based on mammalian cells stably expressing green fluorescent protein (GFP)–labeled Rev protein, which contains a strong heterologous NES [14]. We seeded the reporter cells in 96-well assay plates, incubated them over night at 37°C and treated with LOM612 at a final concentration of 10μM for 30 min. Upon treatment with the nuclear export inhibitor LMB, the GFP-labeled reporter protein accumulated in the cell nucleus (Fig 4B). In contrast, LOM621 failed to induce nuclear accumulation of fluorescence (Fig 4B) indicating that the effect of LOM612 on subcellular FOXO localization is independent of CRM-1.

### Analysis of the NFKB2 translocation upon LOM612 exposure

The activity of many transcription factors such as FOXO, NFAT or NFkB is controlled via the regulation of their subcellular localization. NF-κB proteins shuttle constitutively between the cytoplasm and nucleus, and only accumulate in the nucleus and become activated upon cellular stimulation [6]. IL-1 or TNFα receptor activation induces the degradation of the NF-κB repressor IκBα unmasking the nuclear localization signal (NLS) of NF-κB proteins and promoting its nuclear translocation. To determine if LOM612 compound would lead to indiscriminate accumulation of factors known to be regulated by subcellular localization, we performed immunohistochemical detection of endogenous NFKB2 protein in U2OS cell line using a specific NFKB2 p100/p52 antibody as described before [18]. We showed that LOM612 had no effect on the nuclear export of endogenous NFKB2 transcription factor in this cell line (Fig 4C). In addition, we exposed a cell line that stably expresses the unrelated fluorescent reporter fusion protein nuclear factor of activated T-cells (NFAT-GFP) to 1.5μM LOM612 for 30 min. LOM612 failed to alter the subcellular localization of this protein (data not shown). Taken together these results suggest that LOM612 acts on a molecular target, which specifically regulates the subcellular localization of FOXO proteins.

### Viability of human cell lines in the presence of LOM612

**Fig 5:**
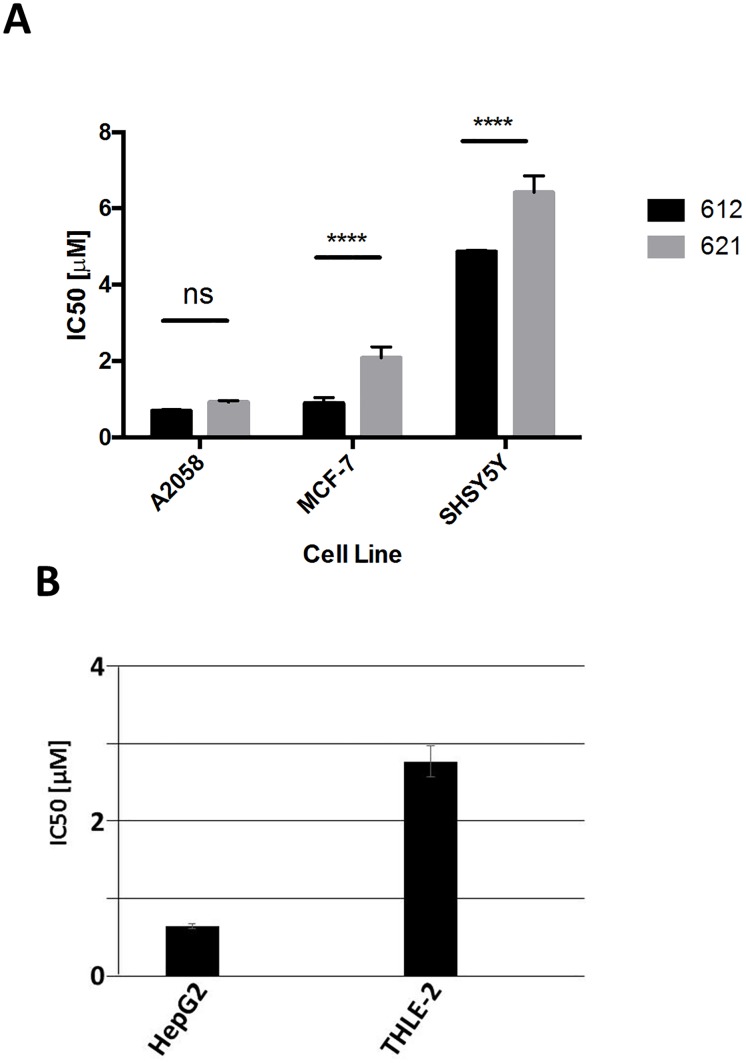
LOM612 compromises the viability of human cancer cell lines. (A) Breast cancer cell line MCF7, melanoma cell line A2058 and the neuroblastoma cell line SH-SY5 were seeded at a concentration of 1× 10^4^ cells/well in 200 μl and treated with compounds LOM612 and LOM621 for 72 hours with eight 2-fold serial dilutions of each compound spanning concentrations from 50μM to 0.39μM. Data is shown as mean ± SEM of three independent experiments. ****P<0.0001 by two-way ANOVA (ns, not significant). (B) Human liver cancer cell lines HepG2 and THLE-2 (cell line derived from primary normal liver epithelial cells) were seeded at a concentration of 1× 10^4^ cells/well in 200 μl and treated with 20 different concentrations of LOM612 from 50μM to 95pM. Data is shown as mean ± SEM of three independent experiments. ****P<0.0001 by two-way ANOVA.

FOXO proteins can orchestrate several transcriptional programs including the onset of apoptosis by enhancing transcription of genes encoding the apoptotic regulators and the induction of cell-cycle arrest by activation the transcription of cyclin-dependent kinase inhibitors [34]. In order to analyze the effect of LOM612 on the viability of human cancer cell lines we performed dose-response analysis after 72 hours of drug exposure. Breast (MCF7), lung cancer (A2058) and glioblastoma cell lines (SHSY5Y) were seeded in 96 multiwell plates and incubated overnight. Cells were treated with twenty different concentrations of LOM612 and LOM621 for 72 hours. LOM612 inhibited the viability of the tested cell lines with IC_50_ values in the high nanomolar or low micromolar range (Fig 5). Treatment of LOM621 also resulted in reduced viability, but was less potent than LOM612. Interestingly, the major difference between the two quinone derivatives was observed after the treatment of MCF7 cells which are known to contain activating mutations within PI3K. These data suggests that LOM612 might selectively counteract FOXO inhibition in cells with increased PI3K/AKT signaling. In order to compare the sensitivity of cancer and non-cancer cells that share a similar biological context to LOM612 treatment, we used well established cellular systems to test hepatcytotoxicity. We exposed HepG2, a human liver cancer cell line commonly used as an in vitro model system for the study of polarized human hepatocytes, and THLE2 cell line derived from primary normal liver epithelial cells and transformed by infection with SV40 large T antigen to several different concentrations of LOM612. Treatment of HepG2 cells with LOM612 resulted in an IC50 value of 0.64μM. Interestingly, non-cancer THLE2 cells were five times less sensitive to LOM612 with an IC50 value of 2.76μM, suggesting that LOM612 might provide a therapeutic window for the treatment of certain human cancers.

## Discussion

Compounds capable of reactivating FOXO based on its tumor suppressor properties are considered a very attractive anti-cancer therapy [6,12,35]. A second area of interest for FOXO activating compounds might be their use to slow aging and to prevent or treat age-related diseases [36,37]. In the present work, we report the discovery of a new compound that specifically induces the nuclear translocation of FOXO. In an attempt to prepare derivatives of Aulosirazole, we identified and synthesized LOM612 –a bioactive compound. Aulosirazole was isolated from the blue-green alga Aulosira fertilissima and is characterized by an unusual isothiazolonaphthoquinone structure [28]. The authors of this work performed corbett assay to show that Aulosirazole exhibits selective toxicity towards solid tumours. The biological activity and unique chemical structure of aulosirazole prompted interest in the chemical synthesis of derivatives which aims to identify antineoplastic agents [38]. We identified LOM612 in an image-based high content screening assay, which monitored the subcellular localization of FOXO proteins. Similar approaches have been used successfully to identify drug candidates for the treatment of human tumours in which FOXO tumour suppressors are inactivated [12,13,39]. LOM612 induced FOXO nuclear translocation with an EC_50_ value in the high nanomolar range indicating that the level of potency is an excellent starting point for a lead optimization process. The observation that a minor structural change, namely the replacement of a N(Me)_2_ moiety with a NH_2_ (**1b**) or a phenyl group (**1c**) at position 3 renders the compound inactive in the FOXO translocation assay, suggests that the dimethylamino group plays an essential role in the interaction with the molecular target. In order to further explore the structural requirements, we are currently preparing a collection of chemical derivatives to perform structure activity relationship analysis. The functional readout for these experiments is based on FOXO subcellular translocation. However, because FOXO proteins are transcription factors that are considered poorly druggable targets [40] it is unlikely that FOXO proteins are the direct molecular targets of LOM612. Modern drug development is increasingly focused on targeted strategies and as a consequence the identification of the molecular target of a bioactive compound is of key importance [41]. The mode of action of Aulosirazole and its derivatives is mostly unknown. Interestingly, the immunoregulatory enzyme indoleamine-2,3-dioxygenase (IDO) has been identified as a potential target of aulosirazole. Furthermore, Pronqodine A, a structurally related natural product isolated from the soil bacterium Streptomyces sp. MK832-95F2 has been shown to be an inhibitor of prostaglandin release [27]. Importantly, several quinones have been identified as selective inhibitors of Cdc25 phosphatases [42–46] which are known to dephosphorylate and in turn activate cyclin-dependent kinases (Cdks) [47]. Intriguingly, FOXO1 has been reported to be a substrate of Cdk2 [48]. In line with these findings, Feng *et al*. showed that Cdc25 regulates the FOXO target gene Matrix Metalloprotein 1 (MMP1) via Cdk2 and FOXO1 [49]. However, it remains to be determined if Cdc25/Cdk2 is also capable of regulating FOXO3a. Mode of action analysis for LOM612 should include the determination of its inhibitory activity in an *in vitro* Cdc25 phosphatase assay. The subcellular localization of FOXO proteins is regulated by a variety of upstream proteins, most prominently serine/threonine kinases. Therefore among the primary target candidates of LOM612 are kinases that are known to phosphorylate FOXOs at sites involved in the regulation of its intracellular localization either directly, such as AKT1–3, SGK 1–3, MST-1, CK1, Cdk2, DKYR1a and IKKβ or indirectly by activating AKT, such as PI3K and PDK1 [5,50]. Some of these enzymes also regulate nuclear translocation of NFKB2 and hence their inhibition would also result in the accumulation of this transcription factor [6]. Accordingly, the analysis of LOM612 or an optimized derivative should include a screening against a selected panel of protein kinases. It is important to note that, the short drug exposure time used in the primary translocation assay precludes any indirect mechanisms that rely on gene transcription or protein synthesis. Importantly, a more detailed study of the toxicity of LOM612 using a broader panel of cancer cell lines would shed light on the molecular profiles that confer cellular sensitivity. The FOXO-inactive analogue LOM621 is an excellent tool to identify cancer cell killing specific to FOXO activation. It is evident that these quinone derivatives are cytotoxic independent of FOXO activation in some cell lines. It is important to note that we observed the strongest FOXO-dependent effect of LOM612 in the MCF7 breast cancer cell line, which contains an oncogenic missense mutation in PIK3CA. This genetic alteration increases the catalytic activity of PI3K, activating the downstream oncokinase AKT that in turn inactivates FOXO supporting the hypothesis that LOM612 mediates part of its cytotoxic effect through FOXO. An important caveat of the study here presented is that this FOXO-specific toxicity of LOM612 is based on limited experimental evidence. In order to translate LOM612 into a therapeutically useful drug candidate, a detailed characterization of its mode of action and an analysis of the molecular profile that sensitizes cells to its cytotoxicity will be of foremost importance. LOM612 or optimized derivatives could be developed to counteract FOXO inactivation in human tumors with constitutively active PI3K/AKT signaling, or to activate cellular defense mechanisms downstream of FOXO factors to slow age-related processes [36,37].

## Supporting Information

S1 Fig LOM612 induces the expression of FOXO target genes. U2OS cells were treated either with DMSO or 5μM of compound LOM612 for 6 hours. qRT-PCR from U2OS cells of FOXO target genes, p27 (A) and FasL (B) whose LOM612-induced expression is induced. Expression relative to GAPDH. n = 2, *p < 0.05.
(PPTX)

## References

[1] Huang H, Tindall DJ. Dynamic FoxO transcription factors. Journal of Cell Science. 2007;120: 2479–2487. 10.1242/jcs.001222 17646672

[2] Webb AE, Brunet A. FOXO transcription factors: key regulators of cellular quality control. Trends Biochem Sci. 2014 ed. 2014;39: 159–169. 10.1016/j.tibs.2014.02.003 24630600 PMC4021867

[3] Zanella F, Link W, Carnero A. Understanding FOXO, new views on old transcription factors. Curr Cancer Drug Targets. 2010 ed. 2010;10: 135–146. 20088800 10.2174/156800910791054158

[4] Eijkelenboom A, Burgering BM. FOXOs: signalling integrators for homeostasis maintenance. Nat Rev Mol Cell Biol. 2013;14: 83–97. 10.1038/nrm3507 23325358

[5] Calnan DR, Brunet A. The FoxO code. Oncogene. 2008 ed. 2008;27: 2276–2288. 10.1038/onc.2008.21 18391970

[6] Zanella F, Santos Dos NR, Link W. Moving to the core: spatiotemporal analysis of Forkhead box O (FOXO) and nuclear factor-kappaB (NF-kappaB) nuclear translocation. Traffic. 2013;14: 247–258. 10.1111/tra.12034 23231504

[7] Brunet A, Bonni A, Zigmond MJ, Lin MZ, Juo P, Hu LS, et al Akt promotes cell survival by phosphorylating and inhibiting a Forkhead transcription factor. Cell. 1999 ed. 1999;96: 857–868. 10102273 10.1016/s0092-8674(00)80595-4

[8] Paik JH, Kollipara R, Chu G, Ji H, Xiao Y, Ding Z, et al FoxOs are lineage-restricted redundant tumor suppressors and regulate endothelial cell homeostasis. Cell. 2007;128: 309–323. 10.1016/j.cell.2006.12.029 17254969 PMC1855089

[9] Hill R, Kalathur RK, Callejas S, Colaco L, Brandao R, Serelde B, et al A novel phosphatidylinositol 3-kinase (PI3K) inhibitor directs a potent FOXO-dependent, p53-independent cell cycle arrest phenotype characterized by the differential induction of a subset of FOXO-regulated genes. Breast Cancer Res. 2014;16: 482 10.1186/s13058-014-0482-y 25488803 PMC4303209

[10] Martins R, Lithgow GJ, Link W. Long live FOXO: unraveling the role of FOXO proteins in aging and longevity. Aging Cell. 2015. 10.1111/acel.12427 PMC4783344 26643314

[11] Willcox BJ, Donlon TA, He Q, Chen R, Grove JS, Yano K, et al FOXO3A genotype is strongly associated with human longevity. Proc Natl Acad Sci USA. National Acad Sciences; 2008;105: 13987–13992. 10.1073/pnas.0801030105 PMC2544566 18765803

[12] Kau TR, Schroeder F, Ramaswamy S, Wojciechowski CL, Zhao JJ, Roberts TM, et al A chemical genetic screen identifies inhibitors of regulated nuclear export of a Forkhead transcription factor in PTEN-deficient tumor cells. Cancer Cell. 2003;4: 463–476. 14706338 10.1016/s1535-6108(03)00303-9

[13] Zanella F, Rosado A, García B, Carnero A, Link W. Chemical genetic analysis of FOXO nuclear-cytoplasmic shuttling by using image-based cell screening. Chembiochem. 2008;9: 2229–2237. 10.1002/cbic.200800255 18756565

[14] Zanella F, Rosado A, Blanco F, Henderson BR, Carnero A, Link W. An HTS approach to screen for antagonists of the nuclear export machinery using high content cell-based assays. Assay Drug Dev Technol. 2007;5: 333–341. 10.1089/adt.2007.058 17638533

[15] Shi F, Smith MR, Maleczka RE. Aromatic borylation/amidation/oxidation: a rapid route to 5-substituted 3-amidophenols. Org Lett. American Chemical Society; 2006;8: 1411–1414. 10.1021/ol060207i 16562904

[16] Pham C-D, Weber H, Hartmann R, Wray V, Lin W, Lai D, et al New cytotoxic 1,2,4-thiadiazole alkaloids from the ascidian Polycarpa aurata. Org Lett. American Chemical Society; 2013;15: 2230–2233. 10.1021/ol400791n 23582084

[17] Howe RK, Gruner TA, Carter LG, Black LL, Franz JE. Cycloaddition reactions of nitrile sulfides with acetylenic esters. Synthesis of isothiazolecarboxylates. J Org Chem. 1978;43: 3736–3742.

[18] Zanella F, Rosado A, García B, Carnero A, Link W. Using multiplexed regulation of luciferase activity and GFP translocation to screen for FOXO modulators. BMC Cell Biol. 2009 ed. 2009;10: 14 10.1186/1471-2121-10-14 19243599 PMC2651847

[19] Link W, Oyarzabal J, Serelde BG, Albarrán MI, Rabal O, Cebria A, et al Chemical interrogation of FOXO3a nuclear translocation identifies potent and selective inhibitors of phosphoinositide 3-kinases. Journal of Biological Chemistry. 2009;284: 28392–28400. 10.1074/jbc.M109.038984 19690175 PMC2788888

[20] Nakae K, Adachi H, Sawa R, Hosokawa N, Hatano M, Igarashi M, et al A simple statistical parameter for use in evaluation and validation of high throughput screening assays. J Biomol Screen. 1999;4: 67–73. 10838414 10.1177/108705719900400206

[21] Link W, Oyarzabal J, Serelde BG, Albarran MI, Rabal O, Cebriá A, et al Chemical interrogation of FOXO3a nuclear translocation identifies potent and selective inhibitors of phosphoinositide 3-kinases. Journal of Biological Chemistry. 2009;284: 28392–28400. 10.1074/jbc.M109.038984 19690175 PMC2788888

[22] Granda TG, Cebrián D, Martínez S, Anguita PV, López EC, Link W, et al Biological characterization of ETP-46321 a selective and efficacious inhibitor of phosphoinositide-3-kinases. Invest New Drugs. 2013;31: 66–76. 10.1007/s10637-012-9835-5 22623067

[23] Mori M, Vignaroli G, Cau Y, Dinic J, Hill R, Rossi M, et al Discovery of 14-3-3 protein-protein interaction inhibitors that sensitize multidrug-resistant cancer cells to doxorubicin and the Akt inhibitor GSK690693. ChemMedChem. 2014;9: 973–983. 10.1002/cmdc.201400044 24715717

[24] Tarrado-Castellarnau M, Cortes R, Zanuy M, Tarrago-Celada J, Polat IH, Hill R, et al Methylseleninic acid promotes antitumour effects via nuclear FOXO3a translocation through Akt inhibition. Pharmacol Res. 2015. 10.1016/j.phrs.2015.09.009 PMC4850087 26375988

[25] Cortes R, Tarrado-Castellarnau M, Talancon D, Lopez C, Link W, Ruiz D, et al A novel cyclometallated Pt(II)-ferrocene complex induces nuclear FOXO3a localization and apoptosis and synergizes with cisplatin to inhibit lung cancer cell proliferation. Metallomics. 2014;6: 622–633. 10.1039/c3mt00194f 24492855

[26] Zanella F, Lorens JB, Link W. High content screening: seeing is believing. Trends Biotechnol. 2010 ed. 2010;28: 237–245. 10.1016/j.tibtech.2010.02.005 20346526

[27] Nakae K, Adachi H, Sawa R, Hosokawa N, Hatano M, Igarashi M, et al NAD(P)H Quinone Oxidoreductase 1 (NQO1)-Bioactivated Pronqodine A Regulates Prostaglandin Release from Human Synovial Sarcoma Cells. j nat prod. 2013;76: 510–515. 10.1021/np300643f 23425216

[28] Stratmann K, Belli J, Jensen CM, Moore RE, Patterson GML. Aulosirazole, a novel solid tumor selective cytotoxin from the blue-green alga Aulosira fertilissima. J Org Chem. 1994;59: 6279–6281.

[29] Ross John F C D W MC C J, Paton RM. Nitrile sulfides Part 16. Synthesis of 1,2-benzisothiazoles via nitrile sulfide cycloaddition reactions. arkivoc. 2013;2013: 372–17.

[30] Paton RM, Ross JF, Crosby J. 1,3-Dipolar cycloadditions of nitrile sulphides to 1,4-quinones: a route to novel isothiazolonaphthoquinones and bis-(isothiazolo)benzoquinones. J Chem Soc, Chem Commun. 1980;: 1194–2.

[31] Hogan IT, Sainsbury M. The synthesis of dendrodoine, 5-[3-(N,N-dimethylamino- 1,2,4-thiadiazolyl]-3-indolylmethanone, a metabolite of the marine tunicate dendroda grossular. Tetrahedron. Pergamon; 1984;40: 681–682.

[32] Sanders MJ, Grunwell JR. Some 1,3-dipolar cycloaddition reactions of nitrile N-sulfides with acetylenes and olefins. J Org Chem. 1980;45: 3753–3756.

[33] Reese CB, Wu Q. Conversion of 2-deoxy- d-ribose into 2-amino-5-(2-deoxy-β- d-ribofuranosyl)pyridine, 2′-deoxypseudouridine, and other C-(2′-deoxyribonucleosides). Org Biomol Chem. 2003;1: 3160–3172. 14527147 10.1039/b306341k

[34] Dansen TB, Burgering BM. Unravelling the tumor-suppressive functions of FOXO proteins. Trends Cell Biol. 2008 ed. 2008;18: 421–429. 10.1016/j.tcb.2008.07.004 18715783

[35] Kau TR, Way JC, Silver PA. Nuclear transport and cancer: from mechanism to intervention. Nat Rev Cancer. 2004;4: 106–117. 10.1038/nrc1274 14732865

[36] Blagosklonny MV. Common drugs and treatments for cancer and age-related diseases: revitalizing answers to NCI's provocative questions. Oncotarget. 2013 ed. 2012;3: 1711–1724. 10.18632/oncotarget.890 23565531 PMC3681506

[37] Martins R K DJ J LG. Targeting FOXOs to slow aging. Molecular Inhibitors in Targeted Therapy. 2015;: 43.

[38] Blunt CE, Torcuk C, Liu Y, Lewis W, Siegel D, Ross D, et al Synthesis and Intracellular Redox Cycling of Natural Quinones and Their Analogues and Identification of Indoleamine-2,3-dioxygenase (IDO) as Potential Target for Anticancer Activity. Angew Chem Int Ed Engl. 2015;54: 8740–8745. 10.1002/anie.201503323 26096359

[39] Link W, Oyarzabal J, Serelde BG, Albarrán MI, Rabal O, Cebria A, et al Chemical interrogation of FOXO3a nuclear translocation identifies potent and selective inhibitors of phosphoinositide 3-kinases. Journal of Biological Chemistry. 2009 ed. 2009;284: 28392–28400. 10.1074/jbc.M109.038984 19690175 PMC2788888

[40] Patel MN, Halling-Brown MD, Tym JE, Workman P, Al-Lazikani B. Objective assessment of cancer genes for drug discovery. Nat Rev Drug Discov. 2013;12: 35–50. 10.1038/nrd3913 23274470

[41] Ferreira BI, Hill R, Link W. Special Review: Caught in the Crosshairs: Targeted Drugs and Personalized Medicine. Cancer J. 2015;21: 441–447. 10.1097/PPO.0000000000000161 26588674

[42] Lazo JS, Aslan DC, Southwick EC, Cooley KA, Ducruet AP, Joo B, et al Discovery and biological evaluation of a new family of potent inhibitors of the dual specificity protein phosphatase Cdc25. J Med Chem. 2001;44: 4042–4049. 11708908 10.1021/jm0102046

[43] Lazo JS, Nemoto K, Pestell KE, Cooley K, Southwick EC, Mitchell DA, et al Identification of a potent and selective pharmacophore for Cdc25 dual specificity phosphatase inhibitors. Mol Pharmacol. 2002;61: 720–728. 11901209 10.1124/mol.61.4.720

[44] Brisson M, Nguyen T, Vogt A, Yalowich J, Giorgianni A, Tobi D, et al Discovery and characterization of novel small molecule inhibitors of human Cdc25B dual specificity phosphatase. Mol Pharmacol. American Society for Pharmacology and Experimental Therapeutics; 2004;66: 824–833. 10.1124/mol.104.001784 15231869

[45] Peyregne VP, Kar S, Ham SW, Wang M, Wang Z, Carr BI. Novel hydroxyl naphthoquinones with potent Cdc25 antagonizing and growth inhibitory properties. Molecular Cancer Therapeutics. American Association for Cancer Research; 2005;4: 595–602. 10.1158/1535-7163.MCT-04-0274 15827333

[46] Brisson M, Foster C, Wipf P, Joo B, Tomko RJ, Nguyen T, et al Independent mechanistic inhibition of cdc25 phosphatases by a natural product caulibugulone. Mol Pharmacol. American Society for Pharmacology and Experimental Therapeutics; 2007;71: 184–192. 10.1124/mol.106.028589 17018577

[47] Hoffmann I, Karsenti E. The role of cdc25 in checkpoints and feedback controls in the eukaryotic cell cycle. J Cell Sci Suppl. 1994;18: 75–79. 7883797 10.1242/jcs.1994.supplement_18.11

[48] Huang H, Regan KM, Lou Z, Chen J, Tindall DJ. CDK2-dependent phosphorylation of FOXO1 as an apoptotic response to DNA damage. Science. American Association for the Advancement of Science; 2006;314: 294–297. 10.1126/science.1130512 17038621

[49] Feng X, Wu Z, Wu Y, Hankey W, Prior TW, Li L, et al Cdc25A regulates matrix metalloprotease 1 through Foxo1 and mediates metastasis of breast cancer cells. Molecular and Cellular Biology. American Society for Microbiology; 2011;31: 3457–3471. 10.1128/MCB.05523-11 PMC3147788 21670150

[50] Van Der Heide L. P., MF Hoekman, Smidt MP. The ins and outs of FoxO shuttling: mechanisms of FoxO translocation and transcriptional regulation. Biochem J. 2004 ed. 2004;380: 297–309. 10.1042/BJ20040167 15005655 PMC1224192

