# Supplementary figures and images for: Discovery of a Novel, Isothiazolonaphthoquinone-Based Small Molecule Activator of FOXO Nuclear-Cytoplasmic Shuttling
Source: PLoS One. 2016 Dec 9;11(12):e0167491. doi: 10.1371/journal.pone.0167491 (PMC5147912; doi:10.1371/journal.pone.0167491)

## Slide 1
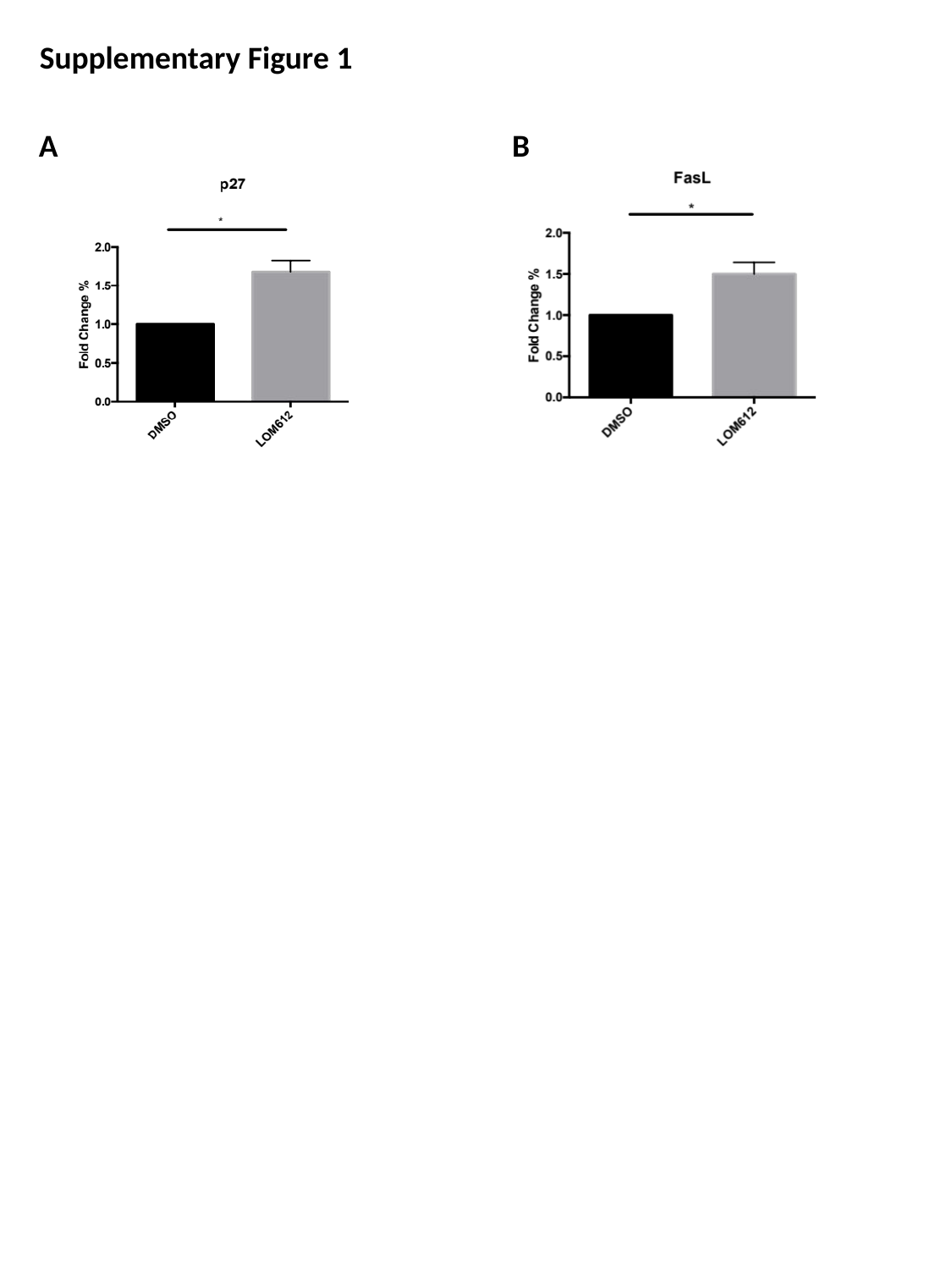

Supplementary Figure 1
A
B

Supplement: S1 Fig — U2OS cells were treated either with DMSO or 5μM of compound LOM612 for 6 hours. qRT-PCR from U2OS cells of FOXO target genes, p27 (A) and FasL (B) whose LOM612-induced expression is induced. Expression relative to GAPDH. n = 2, *p < 0.05. (PPTX) [file pone.0167491.s001.pptx]
